# Genome-Wide Off-Target Analysis in CRISPR-Cas9 Modified Mice and Their Offspring

**DOI:** 10.1534/g3.119.400503

**Published:** 2019-09-06

**Authors:** Yan Dong, Haimei Li, Liang Zhao, Peter Koopman, Feng Zhang, Johnny X. Huang

**Affiliations:** * Gansu Provincial Maternity and Child Care Hospital, 143 North Road, Qilihe District, Lanzhou 730050, China,; † School of Bioscience and Technology, Weifang Medical University, Weifang, Shandong 261053, China, and; ‡ Institute for Molecular Bioscience, The University of Queensland, Brisbane, QLD 4072, Australia

**Keywords:** CRISPR-Cas9, Off-target, Whole-genome sequencing, Genome editing

## Abstract

The emergence of the CRISPR-Cas9 system has triggered a technical revolution in mammalian genome editing. Compared to traditional gene-targeting strategies, CRISPR-Cas9 technology offers a more efficient and cost-effective approach for generating genetically modified animal models. However, off-target cleavage in CRISPR-mediated genome editing is a major concern in the analysis of phenotypes as well as the selection of therapeutic targets. Here, we analyzed whole-genome sequencing (WGS) data from two knock-out (KO) mouse strains generated by using the CRISPR-Cas9 system targeting the *Mmd* and *Paqr8* loci. A total of nine individuals were sequenced including two parents, four F1 offspring and three uninjected control mice. Using GATK and bcftools software, we identified two off-target events in the founder mice. The two CRISPR-Cas9-induced off-target events were predictable using Cas-OFFinder and were not passed on to the offspring that we investigated. In addition, our results indicated that the number of CRISPR-Cas9-induced mutations was not statistically distinguishable from the background *de novo* mutations (DNMs).

CRISPR-Cas systems have become the most powerful tools for genome editing and have been used for the modification of genomes in a wide variety of organisms (Hsu *et al.* 2014; Doudna and Charpentier 2014; Hwang *et al.* 2013). Type II CRISPR-Cas9 is the most commonly used genome editing system, involving Cas9 nuclease from *Streptococcus pyogenes* and a 17-20 nt single guide RNA (sgRNA) complementary to a target site (protospacer) adjacent to a 5′-NGG protospacer adjacent motif (PAM) (Jinek *et al.* 2012; Mulepati *et al.* 2014; Shah *et al.* 2013). However, the possibility of inducing off-target mutations has limited the use of CRISPR-Cas9 technology in research and clinical applications (Cho *et al.* 2014; Fu *et al.* 2013; Lessard *et al.* 2017; Scott and Zhang 2017). Many efforts have been made to minimize the off-target effects of CRISPR-Cas systems by designing high-fidelity nucleases such as eSpCas9 (Slaymaker *et al.* 2016), SpCas9-HF1(Kleinstiver *et al.* 2016), HypaCas9 (Chen *et al.* 2017), HeFspCas9 (Kulcsár *et al.* 2017), HiFiCas9 (Vakulskas *et al.* 2018), Sniper Cas9 (Lee *et al.* 2018) and EvoCas9 (Casini *et al.* 2018). It has also been reported that the use of Cas9 nickase (Trevino and Zhang 2014; Shen *et al.* 2014; Ran *et al.* 2013) or Cas9 from *Streptococcus canis* (ScCas9) (Chatterjee *et al.* 2018) can decrease off-target effects to a certain extent. Furthermore, properly timed addition of Cas9 inhibitors could reduce off-target editing (Harrington *et al.* 2017; Shin *et al.* 2017b). A recent report suggested that precise editing mainly depends on the fourth nucleotide upstream of the PAM (Chakrabarti *et al.* 2019), which could be used in sgRNA design to decrease off-target effects. Nevertheless, the mechanism responsible for CRISPR-Cas-mediated off-target effects remains unclear, and the detection of unintended off-target mutations is of concern.

Unbiased genome-wide methods for detecting DNA double-strand breaks (DSBs) and identifying off-target cleavage events have been established. These methods include BLESS (Crosetto *et al.* 2013), Digenome-seq (Kim *et al.* 2015, 2016), GUIDE-seq (Tsai *et al.* 2015), HTGTS (Frock *et al.* 2015), CIRCLE-seq (Tsai *et al.* 2017) and VIVO (Akcakaya *et al.* 2018). A new method for detecting *in vivo* off-target effects referred to as DISCOVER-seq (Wienert *et al.* 2019) has been reported recently. Furthermore, the analysis of editing fidelity could be more precise if pedigree-matched controls are included (Iyer *et al.* 2018). These methods show high sensitivity in detecting CRISPR-Cas9-induced off-target effects; however, they have limitations such as abundant false positives, cellular model restriction and complex experimental designs.

Here, we assessed off-target events in CRISPR-Cas9-modified knock-out (KO) mice and their offspring by whole-genome sequencing (WGS) analysis and using GATK and bcftools. As part of a broader program of studying the regulation of sex development, we are interested in exploring the developmental roles of several candidate genes obtained from an expression screen, including *Mmd* and *Paqr8* (Zhao *et al.* 2018), via CRISPR-induced loss-of-function experiments, which provide an opportunity for the in-depth study of many technical issues, such as the type and frequency of off-target events in CRISPR experiments. We sequenced nine individual mice, including two CRISPR-Cas9 edited *Mmd* and *Paqr8* KO mice (founders) and their offspring (F1), together with three uninjected controls. We confirmed one CRISPR-Cas9-induced off-target event in each CRISPR-Cas9-edited KO mouse, which all occurred at sites predicted during sgRNA design. We also confirmed that the off-target events identified in parent mice were not passed on to the next generation. More importantly, we found that the frequency of CRISPR-mediated mutagenesis was not statistically distinguishable from the background rate of *de novo* mutations (DNMs).

## Materials and Methods

### Production of genetically modified mice

SgRNAs were selected using the online CRISPR design tool CRISPOR (Haeussler *et al.* 2016) (http://crispor.tefor.net/). Genetically modified mice were produced using a previously described method (Yang *et al.* 2014). In brief, C57BL/6 female mice and CD1 mouse strains were used as embryo donors and foster mothers, respectively. Cas9 protein, tracrRNA and crRNAs were purchased from Integrated DNA Technologies, Singapore. To prepare the Cas9 ribonucleoprotein (RNP), crRNA and tracrRNA were annealed to form an RNA duplex, which was subsequently mixed with Cas9 protein, followed by incubation at 37° for 15 min. For microinjection, the Cas9 RNP containing Cas9 protein (30 ng/µL) and RNA duplexes (10 ng/µL each) was injected into the pronuclei of zygotes. The injected zygotes were cultured overnight in EmbryoMax Advanced KSOM medium (Merck Millipore, MR-101-D) at 37° under 5% CO_2_ to the two-cell stage. Then, 16-20 two-cell-stage embryos were transferred to the oviducts of pseudopregnant CD1 females at 0.5 dpc. All animal procedures were conducted at the University of Queensland and were approved by the Animal Care Committee at the University of Queensland Animal Ethics Committee.

### WGS, variant calling and filtration

Whole-genome sequencing libraries of nine samples were prepared using standard protocols for the Illumina X10 platform, generating 2,492 gigabytes of raw data. The Illumina raw reads were trimmed using Cutadapt (Martin 2011) to remove adaptors and bases of low quality. Then, the cleaned reads were mapped to the reference genome from the Ensembl database (http://ftp.ensemblorg.ebi.ac.uk/pub/release-89/) using the BWA mem program (Li and Durbin 2009). Sequence variants were called for all parents and offspring using GATK and bcftools. Variant calling and filtration using GATK software were performed with the UnifiedGenotyper and VariantFiltration commands, respectively. The parameters for single-nucleotide variants (SNVs) were set as follows; QD < 2.0, FS > 60.0, MQ < 40.0, MQRankSum < -12.5, ReadPosRankSum < -8.0, and those for indels were QD < 2.0, FS > 200.0, ReadPosRankSum < -20.0. The bcftools version and command options were as follows: bcftools-1.9 mpileup, bcftools mpileup –Ou, bcftools call –cv, bcftools norm –f, bcftools filter -Oz -s LOWQUAL -e “QUAL < 10 or DP < 10”. Bcftools mpileup configured for sensitivity required a minimum allelic fraction of 0.05. Bcftools called between 220,709 and 252,140 variants per sample with a genotyping quality ≥10 and a median of 245,475 variants. All variant loci were required to have a total depth of at least 10 reads for further analysis. DNM calling for all variants was performed independently for each parent/offspring. The following criteria were applied to remove false positives and obtain final variants: (1) A minimum variant allele fraction of 5% was set to allow mosaic alleles. (2) Any variant coincident with an allele reported in the C57BL/6NJ strain in the Mouse Genomes Project was removed. (3) All individual repeat tracks within the University of California Santa Cruz (UCSC) RepeatMasker track (approximately 1.2 Gb) were merged, and all variants inside or 1 bp adjacent to merged repeats were removed. (4) Any *de novo* variant shared by two or more samples was removed.

### Data Availability

The WGS data used in this study can be accessed at http://gsa.big.ac.cn/, with the accession of CRA001780. All data necessary for confirming the conclusions of the article are present within the article, figures, tables and supplementary tables. Supplemental material available at FigShare: https://doi.org/10.25387/g3.8137763.

## Results

### Targeting of Mmd and Paqr8 genes

**Figure 1:**
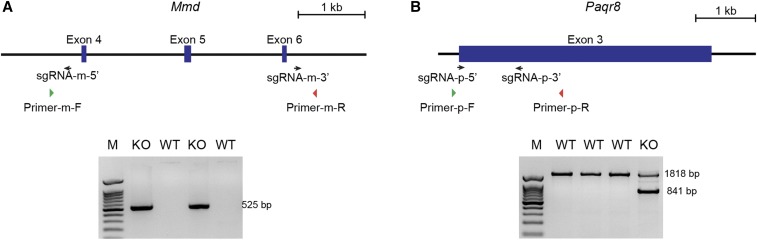
Generation of *Mmd* and *Paqr8* KO alleles using CRISPR/Cas9. (A) Targeting strategy of the *Mmd* gene. PCR genotyping primers indicated in the scheme produced bands with correct size (525 bp) in targeted mice, but not in WT samples. (B) Targeting strategy of the *Paqr8* gene. PCR genotyping primers indicated in the scheme produced a band of 841 bp in KO allele, and 1818 bp in WT allele. Black arrows indicate sgRNAs; Green and Red arrows indicate PCR primers; M, DNA marker; KO, knock-out samples; WT, wild type samples.

To generate *Mmd* or *Paqr8* KO alleles, we employed a pair of sgRNAs to delete one or more coding exons in each gene. The two sgRNAs (sgRNA-m-5′ and sgRNA-m-3′) used for disrupting the *Mmd* gene were located in introns 3 and 6, respectively, resulting in the deletion of a 3448 bp region including exons 4-6 (Figure 1A). In the targeting of *Pagr8*, both sgRNAs (sgRNA-p-5′ and sgRNA-p-3′) were located within exon 3, and a 977 bp region was deleted (Figure 1B).

The Cas9 protein and the sgRNAs targeting *Mmd* or *Papr8* were coinjected into 40 C57BL/6 mouse zygotes via pronuclear microinjection. The injected zygotes were allowed to develop to the 2-cell stage and were then transferred into 0.5-day postcoital pseudopregnant CD1 females. After natural delivery, a total of three founder mice with the desired mutant alleles were identified using PCR and Sanger sequencing (Table 1). The primers used for PCR genotyping are shown in Supplementary Table 1. We then tested germline transmission by backcrossing male founder mice with wild-type (WT) C57BL/6 females and successfully generated heterozygous F1 individuals.

**Table 1:** CRISPR/Cas9-mediated gene targeting in C57BL/6 mice

Gene	Injected Zygotes	Transferred 2-cell embryos (Recipients)	Newborns	Mouse with correct mutant allele(s)
*Mmd*	40	34 (2)	4	2
*Paqr8*	40	38 (2)	4	1

### WGS data analysis

**Figure 2:**
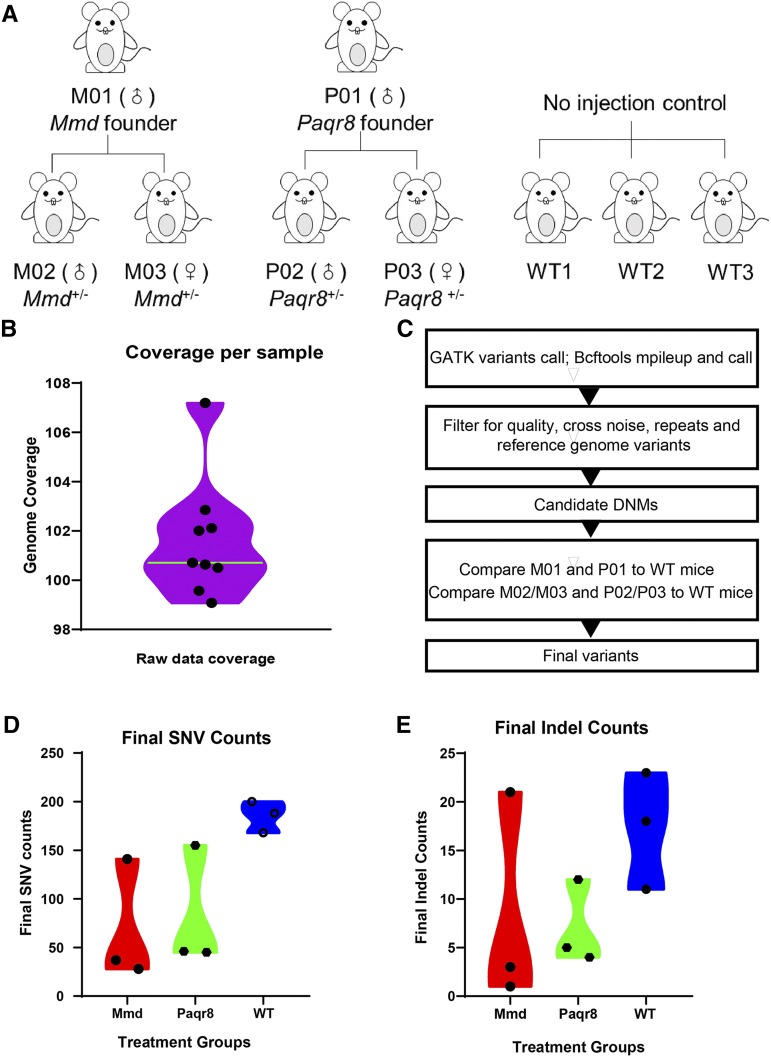
Analysis of CRISPR-Cas9-induced off-target events using WGS. (A) Mice used in this study. (B) WGS coverage. Nine mice were subjected to WGS, yielding a median depth of 101.6X. (C) Work flow of variant calling and filtering. (D) Final SNV counts summary. (E) Final indel counts summary.

We sequenced the whole genomes of nine mice (Figure 2A), including two founder mice (M01 and P01), four heterozygous mice (M02, M03, P02 and P03) and three uninjected control mice (WT1, WT2 and WT3). Sequencing was performed on the Illumina X10 WGS platform, yielding a median sequencing depth of 101.6x per genome (Figure 2B). We first normalized the WGS data by deleting regions of low-quality reads using Cutadapt software, setting the threshold at 30 (Martin 2011). Unclear reads and fragments of less than 60 bp were also deleted from the data. After data processing, we acquired an average of 241.32 GB of clean data for each sample. The work flow of variant calling and filtering is indicated in Figure 2C. We performed variant calling using both GATK (UnifiedGenotyper and VariantFiltration) software (McKenna *et al.* 2010) and bcftools (mpileup and bcftools call) (Li 2011), resulting in median values of 240,156 variants per sample (129,970 SNVs/110,186 indels) and 238,913 variants per sample (127,508 SNVs/111,405 indels), respectively (Table 2). We then filtered the high-quality calls using reference mouse genome GRCm38 (Ensembl), the UCSC database and the International Mouse Phenotyping Consortium (IMPC) database for all samples, removing repeats and coincident regions. After visual inspection of the filtered variants, false positives were removed, and candidate DNMs for each sample were acquired (Table 2). As a result, median values of 1020 candidate DNMs (708 SNVs/312 indels) and 470 candidate DNMs (312 SNVs/158 indels) were obtained using the GATK method and bcftools, respectively. Furthermore, we compared the candidate DNMs of M01 and P01 mice to those of three WT mice and removed coincident mutations. Similarly, in the case of F1 offspring mice (M02, M03, P02 and P03), we removed the coincident mutations found in WT mice. Final SNV and indel counts were then obtained (Figure 2D and 2E). As a result, the median number of final SNVs was 141, and the median number of final indels was 11 (Table 2).

**Table 2:** Summary of high-quality variant counts, filtered DNMs and final variants

Mouse sample	Treatment Group	Relationship	Quality passed variants (GATK)		Quality passed variants (bcftools)		Candidate *de novo* mutations (GATK)		Candidate *de novo* mutations (bcftools)		Final SNVs	Final Indels
			SNV	Indel	SNV	Indel	SNV	Indel	SNV	Indel		
M01	Cas9 RNP	Parent	129169	113307	130516	117545	325	165	647	301	141	21
M02		Offspring	128666	116517	126096	123203	203	100	464	231	37	1
M03		Offspring	130771	114722	127508	120029	189	91	464	229	28	3
P01	Cas9 RNP	Parent	130152	115350	135322	116818	312	158	708	312	155	12
P02		Offspring	128514	110186	126265	111366	219	105	392	221	46	5
P03		Offspring	129970	102837	126670	94039	197	80	399	162	45	4
WT1			130319	109723	134782	110693	526	351	1447	439	200	23
WT2			128871	109005	124798	109253	366	225	935	320	168	11
WT3			130665	112541	131262	111405	402	311	1064	385	188	18
Median			129970	110186	127508	111405	312	158	647	301	141	11

### Statistical analysis of variants from KO and WT samples

We next conducted a Kruskal-Wallis Rank test using the candidate and final variant counts (Table 3). The results showed no significant differences between the *Mmd*, *Paqr8* and WT groups (*P* = 0.0594 and *P* = 0.06081 for candidate SNV counts and indel counts, respectively; *P* = 0.05091 and *P* = 0.2881 for final SNV counts and indel counts, respectively). Additionally, a Wilcoxon rank test was performed to identify significant differences between the CRISPR-Cas9 edited groups (Table 3). The results showed no significant difference between the two groups, with p values of 0.4 and 0.7 for the final SNV and indel counts, respectively.

**Table 3:** Statistical analysis of final variant counts

Groups compared (mouse number)	Value compared	Test	Result	Null Hypothesis	Reject Null Hypothesis
*Mmd*(3), *Paqr8*(3), WT (3)	Candidate SNV counts	Kruskal-Wallis Rank Sum	(G)chi-squared = 5.4222, df = 2, p-value = 0.06646 (B) chi-squared = 5.6471, df = 2, p-value = 0.0594	No significant difference in treatment group means	No
	Candidate indel counts	Kruskal-Wallis Rank Sum	(G)chi-squared = 3.8222, df = 2, p-value = 0.1479 (B)chi-squared = 5.6, df = 2, p-value = 0.06081	No significant difference in treatment group means	No
	Final SNV	Kruskal-Wallis Rank Sum	chi-squared = 5.9556, df = 2, p-value = 0.05091	No significant difference in treatment group means	No
	Final indels	Kruskal-Wallis Rank Sum	chi-squared = 2.4889, df = 2, p-value = 0.2881	No significant difference in treatment group means	No
*Mmd*(3), *Paqr8*(3)	Final SNV counts	Wilcoxon Rank Sum	W = 2, p-value = 0.4	No significant difference in treatment group means	No
	Final indel counts	Wilcoxon Rank Sum	W = 3, p-value = 0.7	No significant difference in treatment group means	No

### Determination of Cas9-induced off-target effects

To investigate whether our strategy can identify Cas9-induced off-target events, we compared the final variants with the expected off-target effects for each sgRNA predicted by Cas-OFFinder software (Bae *et al.* 2014). A total of 114 off-target sites were predicted for the targeting of the *Mmd* gene (31 and 83 for sgRNA-m-5′ and sgRNA-m-3′, respectively), while 163 loci were predicted for the *Paqr8* gene (55 and 111 for sgRNA-p-5′ and sgRNA-p-3′, respectively) (Supplementary Table 1). We identified one indel in each founder mouse (M01 and P01), which was shown by both our final variants and the predicted lists of Cas-OFFinder, indicating real Cas9-induced off-target events. The indels were located on *chr14* and *chr2* and were generated by sgRNA-m-3′ and sgRNA-p-5′, respectively (Table 4). These mutations might be filtered out among the final variants of the offspring mice using our filtering strategy. Thus, we performed PCR verification and Sanger sequencing to confirm the off-target mutations in the offspring mice. We did not find those mutations in M02, M03, P02 and P03, indicating that the off-target mutant alleles were not passed on to the F1 mice that we inspected.

**Table 4:** Confirmation of CRISPR Cas9-induced off-target events

Gene	Number of predicted off-target mutations	Sample	Real off-target sites	Position
*Mmd*	114	M01	1(indel)	chr14:27199770
		M02	0	
		M03	0	
*Paqr8*	163	P01	1(indel)	chr2:164786352
		P02	0	
		P03	0	

## Discussion

We generated *Mmd* and *Paqr8* KO mouse strains using the CRISPR-Cas9 system. To increase the targeting efficiency, we applied a two-sgRNA system with the aim of deleting multiple exons of the *Mmd* gene and part of exon 1 of the *Paqr8* gene. We performed pronuclear microinjection of the Cas9 protein in our experiments, which may contribute to minimizing off-target effects because the protein can be degraded a few hours after microinjection. We obtained four newborns from 40 injected zygotes for each strain, with up to 50% of the newborn mice harboring biallelic mutations in the targeted genes, suggesting that the two-sgRNA system is highly efficient in generating KO mice. However, the use of two sgRNAs may increase Cas9-induced off-target events. Although no unexpected phenotypes were observed in the founder mice, we conducted WGS analysis to identify off-target sites.

The mechanism responsible for Cas9-induced off-target effects could be highly related to the tolerance of sgRNA mismatches, especially the seed sequence of the sgRNAs, which is the 6-11 nt region immediately upstream of the PAM sequence (Zhang *et al.* 2015; Shin *et al.* 2017a). Moreover, GC contents and the positions of cytosine and adenine are suggested to contribute to Cas9-induced off-target effects (Zhang *et al.* 2015). Thus, most CRISPR design tools score sgRNAs based on the number of nucleotide mismatches, the position and the distance to the PAM, providing potential off-target sites predicted by algorithms. A recent study presented a binding energy model for Cas9 RNP in which energy parameters were introduced into sgRNA design to increase specificity (Alkan *et al.* 2018). Nevertheless, there could be other factors involved in off-target cleavage that have not yet been incorporated into current algorithms. In our study, although the two confirmed off-target sites were both predicted by Cas-OFFinder, there is still a lack of evidence showing the accuracy of current algorithms.

Previous studies have addressed concerns by using WGS for detecting DNMs, which may represent mutations introduced by the CRISPR-Cas9 system (Iyer *et al.* 2015; Mianné *et al.* 2016). To overcome the defects of studies using only strain-matched controls, pedigree-matched controls were introduced for the analysis of off-target events (Iyer *et al.* 2018). Furthermore, a new method referred to as GOTI (Genome-wide Off-target analysis by Two-cell embryo Injection) was developed to detect off-target mutations (Zuo *et al.* 2019). However, these methods require the sequencing of all parents to establish pedigree trees or to perform microinjection at the two-cell stage. In this study, we sequenced the whole genomes of one parent and two offspring from each strain, together with three uninjected mice. We found that the data obtained from those mice were sufficient to analyze off-target events when two widely used analysis software platforms, GATK and bcftools, are used. Variant calling was independently performed using each software platform, and high-quality variants were then filtered using the UCSC and IMPC databases, resulting in candidate DNMs. Final variants were then obtained by comparing the parents and/or controls using the criteria mentioned in the section Materials and Methods. Two off-target sites were successfully identified during the analysis, indicating that a few background controls together with a partial pedigree tree may fulfill the detection of off-target sites induced by CRISPR-Cas9. We have shared our WGS data at Genome Sequence Archive (see Data Availability), which can be utilized by other researchers.

Intriguingly, statistical analysis showed that there was no significant difference between Cas9-induced mutagenesis and background DNMs, which is consistent with previous studies (Iyer *et al.* 2018; Akcakaya *et al.* 2018). In addition, off-target and on-target mutations could segregate through meiosis and homologous recombination, especially when they are located on different chromosomes. The two off-target effects identified in the current study were located on *chr14* and *chr2*, whereas *Mmd* and *Paqr8* are located on *chr11* and *chr1*, respectively. Although the off-target mutations are heritable, we did not find them in the F1 mice that we examined. Nevertheless, it is highly likely that a two-sgRNA system may increase Cas9-induced off-target effects; therefore, it is not recommended to use more than two sgRNAs unless large deletions are being generated.
